# *Caenorhabditis elegans* dauers vary recovery in response to bacteria from natural habitat

**DOI:** 10.1002/ece3.6646

**Published:** 2020-08-24

**Authors:** Louis T. Bubrig, John M. Sutton, Janna L. Fierst

**Affiliations:** ^1^ Department of Biological Sciences The University of Alabama Tuscaloosa AL USA

**Keywords:** bacteria, *Caenorhabditis elegans*, dauer, dormancy, habitat

## Abstract

Many species use dormant stages for habitat selection by tying recovery to informative external cues. Other species have an undiscerning strategy in which they recover randomly despite having advanced sensory systems. We investigated whether elements of a species' habitat structure and life history can bar it from developing a discerning recovery strategy. The nematode *Caenorhabditis elegans* has a dormant stage called the dauer larva that disperses between habitat patches. On one hand, *C. elegans* colonization success is profoundly influenced by the bacteria found in its habitat patches, so we might expect this to select for a discerning strategy. On the other hand, *C. elegans*' habitat structure and life history suggest that there is no fitness benefit to varying recovery, which might select for an undiscerning strategy. We exposed dauers of three genotypes to a range of bacteria acquired from the worms' natural habitat. We found that *C. elegans* dauers recover in all conditions but increase recovery on certain bacteria depending on the worm's genotype, suggesting a combination of undiscerning and discerning strategies. Additionally, the worms' responses did not match the bacteria's objective quality, suggesting that their decision is based on other characteristics.

## Graphical abstract

Some dormant organisms try to sense their external environments to avoid recovering from dormancy in poor conditions. We suspected that the habitat structure and life history of the nematode *Caenorhabditis elegans* would prevent the worm from adjusting its own recovery. We found that *C. elegans* recovers from dormancy in all conditions, even poor ones, but enhances recovery in some situations.

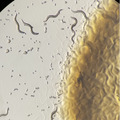

## 1 INTRODUCTION

Many organisms use developmentally arrested dormant stages to endure harsh environments and/or disperse to better ones (Baskin & Baskin, 1998). Dormant organisms must recover to resume growth but this transition is often irreversible and exposes the individual to new dangers (Raimondi, 1988). Therefore, individuals that assess local conditions and tie this information to their recovery can increase their fitness (Keough & Downes, 1982). Unsurprisingly, this has led to the evolution of a diversity of discerning strategies (Baskin & Baskin, 1998) (Johnson, Lewis, Nicols, & Degnan, 1997). The cues that induce dormant stage recovery are tailored to the organism's abiotic and biotic needs; the strategies can be as simple as measuring temperature (Finch‐Savage & Leubner‐Metzger, 2006) or detecting conspecifics (Burke, 1986) and as complicated as parsing out signals from whole communities. Coral larvae, for example, can differentiate between algal species growing in a prospective settlement site (Harrington, Fabricius, De'Ath, & Negri, 2004). While many species develop these discerning strategies, other species seem to adopt an undiscerning strategy, recovering under all conditions, even poor ones (Keough & Downes, 1982). If these species have variable habitat qualities that impact their fitness, why are not discerning strategies being selected for?

**Figure 1:**
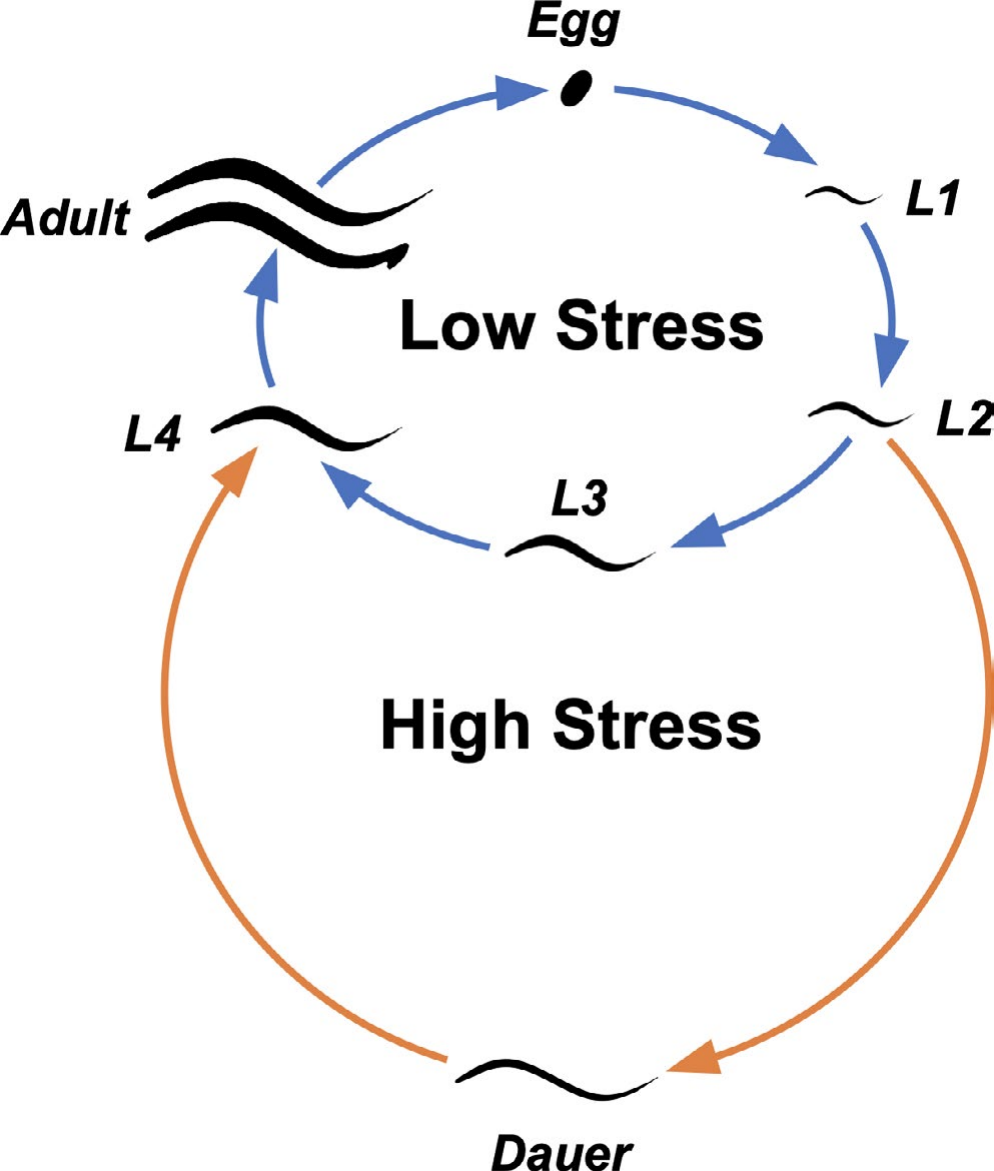
The life cycle of *Caenorhabditis elegans*. Newly hatched worms that sense high environmental stress become dauer larvae instead of the normal third larval stage (L3). Dauers that sense improving conditions can reenter the low stress cycle and continue to adulthood

One possible explanation is that discerning strategies only arise if they help organisms avoid bad habitats and find good ones. A dormant organism may ignore salient information about its environment if it has no capacity to act on it (Raimondi, 1988). Behavioral constraints, life‐history traits, and habitat structure may prevent the development of discerning strategies, even when they would seem useful at first glance. In this project, we investigated how the nematode *Caenorhabditis elegans* recovers from its dormant stage—the dauer (Figure 1)—given that the species seems pulled in two opposite directions. On one hand, the dauer appears perfectly suited for a complex habitat recognition system. This dormant stage is carried by small invertebrates to new habitat patches that vary substantially in their quality with some patches being totally inhospitable due to their bacterial community composition (Samuel, Rowedder, Braendle, Felix, & Ruvkun, 2016) (Kiontke & Sudhaus, 2006). Bacteria can be good sources of food or deadly pathogens depending on the species (Felix & Braendle, 2010) (Samuel et al., 2016), and *C. elegans* can certainly differentiate between them (Johnson et al., 1997), at least from a mechanistic standpoint. Recovering is an irreversible decision that affects fitness: Dauers are hardy and long‐lived but cannot reproduce (Cassada & Russell, 1975; Ellenby, 1968; Klass & Hirsh, 1976), while recovered worms can establish colonies but are vulnerable.

On the other hand, behavioral constraints and habitat structure may keep *C. elegans* from developing discerning recovery strategies. *C. elegans* dauers cannot control their invertebrate carriers and will be dropped off in bad habitats and good habitats alike. Unlike seeds which can stay put and ride out bad conditions for years (Baskin & Baskin, 1998), *C. elegans*'s natural habitats are ephemeral, rotting away in a matter of days (Ferrari et al., 2017). Unlike many marine invertebrates which can reject bad sites and move on to others (Pawlik, 1992), *C. elegans* may be stuck wherever it first arrives; we have no evidence that the same dauer can experience multiple habitats before recovering, although little is known about *C. elegans*’ embarkment and disembarkment in general. External cues are only useful if they are actionable (Raimondi, 1988), so the worms' lack of choice may lead them to ignore these cues in favor of simply recovering indiscriminately in the hopes of establishing a foothold.

We investigated how these opposing aspects of *C. elegans*' ecology translate into recovery strategies by exposing dauers to a range of bacteria. We used four ecologically relevant bacterial species isolated from *C. elegans*' natural habitat (Samuel et al., 2016). Samuel et al. (2016) categorized each bacterial species based on *C. elegans* population growth and immune system activation. *Raoultella* sp. JUb54 and *Providencia* sp. JUb39 are considered "beneficial" because they support *C. elegans* population growth and do not activate the worm's immune system. *Serratia* sp. JUb9 and *Pseudomonas* sp. BIGb0427 are "detrimental" because they are pathogenic and cannot support *C. elegans* populations. We sequenced the genomes of these four species to confirm their identities and help rectify the imbalance between the extensive resources for *C. elegans* and a lack of resources for its natural associates (Frezal & Felix, 2015). In addition to the natural bacteria, we included *Escherichia coli* OP50, the standard laboratory food which is not a natural food source (Frezal & Felix, 2015), and a control treatment with no food at all. To determine whether *C. elegans* exhibits intraspecific variation in dormancy recovery, we tested three different worm strains that are geographically and genetically distinct. N2, isolated in Bristol, is the *C. elegans* reference strain which has been used since the mid‐1900s. CB4856 is a very distant relative isolated in Hawaii. JU1395 is a much more recent isolate taken from France in 2008. We exposed dauers to bacteria for three hours, after which we collected and scored them based on their recovery status. Our data suggest that *C. elegans* dauer recovery has elements of both undiscerning and discerning strategies: *C. elegans* dauers recover regardless of condition but enhance their recovery when detecting certain bacteria. Additionally, *C. elegans* exhibits intraspecific variation in its recovery behavior.

## 2 DISCUSSION

When habitat quality affects an organism's fitness, we expect natural selection to align an organism's recovery with habitat quality. In the case of *C. elegans*, variation in habitat quality might select for worms that can differentiate between bacteria, a key determinant of establishment success. However, *C. elegans* disperses via a carrier and cannot choose its habitat; modulating dauer recovery might not provide worms with any advantage (Raimondi, 1988). In this case, the fittest strategy could be one of high rapid recovery across the board to outcompete other colonists. Our data are consistent with both of these hypotheses.

All three worm strains recovered substantially in all treatments—even in the absence of food—which supports the hypothesis that *C. elegans* cannot choose its habitat and recovers no matter what. Recovery is guaranteed through other signals such as decreasing population density which *C. elegans* measures using a pheromone (Golden & Riddle, 1984). The pheromone's concentration decreases when worms are washed in the lab or when a small number of worms colonize a patch, ensuring some degree of recovery in all new habitats. Presumably, worms that try to colonize a bad habitat have higher fitness than worms that refuse to try at all (Johnson et al., 1997). The basal level of recovery depended on the worm strain. N2 and CB4856 have similarly low recoveries, and CB4856’s relatively low recovery is consistent with its reluctance to enter the dauer stage (Neal et al., 2016). JU1395’s recovery was much higher. More time points are needed to determine whether this order holds; perhaps N2 and CB4856 separate from each other or catch up to JU1395 over time. Regardless, these recovery differences may result from genetic variation in dauer‐controlling pathways, especially in components like pheromone production that control dauer recovery broadly. Among other differences, JU1395 has a polymorphism in *daf‐22*, a gene involved in dauer pheromone synthesis (Golden & Riddle, 1984), while N2 and CB4856 have identical *daf‐22* sequences. Determining these polymorphisms' functional impact—if any—can be addressed in future work using the genetic tools available in *C. elegans*. A transcriptome study could confirm that these genes are expressed during recovery and identify more target genes (Wang & Kim, 2003). We could then determine their functional significance by implementing gene knock‐ins or recombinant lines in more dauer recovery assays. Additionally, we can expand our search for polymorphisms to other gene families, especially changes in the odorant receptors that drive variation in response to specific bacteria (Lee et al., 2019).

From an evolutionary point of view, differences between the strains could reflect varying levels of acceptable risk. Some conditions, such as consistently high levels of pathogens, may favor more cautious strategies with slower recovery, while other conditions select for a faster response. Strategies may also diverge when different strains regularly co‐occur in the same habitat. A strain that frequently encounters a more cautious strain could benefit by recovering rapidly and establishing early. Timing developmental decisions to beat out other strains is not unheard of in nematodes; strains of the related nematode *Pristionchus pacificus* intentionally drive other strains of the same species into the dauer stage to stop them from feeding (Bose et al., 2014).

Dauer recovery differs among the bacterial treatments which is evidence for a more discerning strategy. Interestingly, the species does this in a way that is still consistent with the undiscerning strategy; no response is lower than the control but some bacteria can enhance recovery. Recovery will always occur, even in bad conditions, but can be accelerated upon detecting good conditions. What *C. elegans* interprets as "good," however, is much more complicated than we had assumed. The worms' responses do not simply reflect the objective quality of the bacteria. The most favorable bacteria—that is, the one which elicited the greatest response—differs with worm strain. N2 responds highly to *E. coli* OP50 and so does CB4856, but CB4856 also responds highly to the detrimental bacterium *Serratia* sp. JUb9. In contrast, JU1395 shows little response to *E. coli* OP50 but strongly responds to *Providencia* sp. JUb39. These results indicate a lack of matching between recovery and a bacterium's objective quality. For instance, we demonstrated that *Serratia* sp. JUb9 rapidly kills all three worm strains and does not support growing populations. Despite this, CB4756 and JU1395 unexpectedly have enhanced dauer recovery on the bacterium even though the newly recovered population will fail to grow on it. Similarly, *Providencia* sp. JUb39 is objectively a nutritious food source but CB4856 has reduced recovery on it.

This lack of matching between food quality and response could have several explanations. While the fecundity assay demonstrated that the three strains grew similarly on the bacteria, finer scale fitness assays could reveal slight differences in the strains’ growth. Furthermore, certain combinations of worm strain and bacteria may never occur in nature or have occurred recently enough that selection has not had time to act (Chew, 1977). Imperfect matching could also occur when odorants are shared across many bacterial species, so selection on one worm–bacteria response spills over into other responses. It is possible that worms can glean information about the bacterial community as a whole from interactions with individual species. Perhaps the presence of a specific bacterium in a community signals overall community health, substrate composition, or age of the patch (Johnson et al., 1997); some species of coral, for instance, deduce their depth by sensing the composition of nearby bacterial communities (Webster et al., 2004). Finally, bacteria may release odorants to specifically manipulate bacteriovore behavior. While worms use bacterial cues to detect and avoid bacteria (Meisel, Panda, Mahanti, Schroeder, & Kim, 2014), these bacteria may be under selection to evade detection or, in the case of pathogens, to attract vulnerable hosts. Dauer recovery rates could therefore be influenced by pathogen avoidance and host manipulation. Dauer behavior is known to be manipulated by at least one non‐nematode organism, the beetle *Exomala orientalis* (Cinkornpumin et al., 2014), so manipulation by bacteria is certainly feasible. Interestingly, *Serratia marcescens*, a congener of *Serratia* sp. JUb9, is strongly attractive to *C. elegans* despite its high pathogenicity (Zhang, Lu, & Bargmann, 2005) (Pradel et al., 2007), an observation that has puzzled many researchers.

Despite these patterns, the data from this project exhibit high variability within and among trials. Dauer recovery is controlled by dozens of external and internal signals, many of which cannot be controlled for despite our best efforts to standardize the procedure. Variability within each trial can be partially explained by the pooling process; collecting dauers after two weeks of starvation pools together dauers that entered the stage at different time points and in response to different signals, both of which can alter their propensity to recover (Klass & Hirsh, 1976). Slight differences in timing, incubation temperature, and contamination on starvation plates could all contribute to among‐trial variation by carrying this information through the dauer stage or even passing it along to offspring via transgenerational effects. For instance, we seeded our starvation plates with L1 larvae that had hatched overnight in the absence of food. L1 starvation shifts gene expression—including some dauer‐controlling pathways—in the individual and its offspring (Rechavi et al., 2014), so our dauers’ recovery decisions may have been influenced by this previous environment. Future studies can uncover how predauer conditions influence the bacterial discernment we observed in this experiment.

Our results demonstrate that *C. elegans* dauers modulate their recovery based on the bacteria they detect in their new habitat. If these differences in recovery result from selection, this suggests that tying recovery to external cues still provides some kind of fitness benefit. Assuming that habitat structure bars *C. elegans* dauers from dispersing to a better habitat in time or space, perhaps the variety of strategies results from finer scale fluctuations in habitat quality over the course of the rotting process. Additionally, conspecifics that frequently co‐occur could maintain divergent strategies that vary in their levels of acceptable risk or other characteristics. To understand the breadth of dauer recovery strategies, future projects should incorporate more strains from *C. elegans* and from closely related nematode species. The mechanisms producing these strategies can be narrowed down using mutant or recombinant strains that incorporate changes in dauer‐controlling components. An alternative approach could use the four bacterial genomes presented in this study; suspected bacterial odorant genes could be knocked out, causing dauers to retain or change their recovery response.

Of course, our results could also indicate that our assumption about *C. elegans* was incorrect; future work may reveal that the species has more control over its habitat selection than we thought. Either way, behavioral strategies do not simply evolve in response to strong environmental pressures. A full understanding must take into account an organism's ecological context, habitat structure, and life history, all of which contribute to the evolution of dormancy recovery strategies.

## 3 METHODS AND MATERIALS

### 3.1 Worms and bacteria

The strains of *C. elegans* used for this project were N2, CB4856, and JU1395, which were received from the Caenorhabditis Genetics Center (CGC). N2 is the standard laboratory strain which was isolated in Bristol, UK, in 1951 but not frozen until 1969. CB4856 was isolated in Hawaii in 1972, and JU1395 was isolated in Montsoreau, France, in 2008.

*E. coli* OP50 was also received from the CGC. The four wild bacteria were all isolated from different sites in France between 2004 and 2009 (Samuel et al., 2016). *Providencia* sp. JUb39 and *Raoultella* sp. JUb54 were taken from rotting apples, and *Serratia* sp. JUb9 was found in compost. These three species were acquired from Marie‐Anne Félix at Institute of Biology of the Ecole Normale Supérieure (IBENS). *Pseudomonas* sp. BIGb0427 was isolated from the rotting stem of a butterbur plant and was acquired from Buck Samuel at Baylor College of Medicine. All worms and bacteria were frozen at −80°C, and aliquots thawed for each experimental replicate.

### 3.2 Setting up experimental plates

Approximately three weeks before the experiment, worms of each strain were thawed and placed on 100 mm *E. coli*‐seeded nematode growth medium (NGM) plates (Stiernagle, 2006). These worms were incubated at 20°C and expanded to seven plates per strain over the course of six days. The original thaw plates were discarded, and the remaining six plates per strain were washed with water and the worms bleached using standard laboratory protocols to limit contamination (Stiernagle, 2006). Bleached eggs hatched overnight on a rocker at room temperature. The next day, hatched worms were placed onto six new *E. coli*‐seeded NGM plates per strain. The worms were incubated at 20 º C for two weeks to induce dauer formation via starvation and overcrowding.

Experimental plates were 100‐mm standard NGM plates. Three of these plates were used for the control treatment and contained an addition of 0.1% ampicillin, a broad‐spectrum antibiotic used to prevent bacterial growth. Plates were assigned random number IDs to blind the experiment and ensure unbiased counting later on. Five bottles of 50 ml Luria Broth were inoculated with each of the five bacterial species, and a sixth control bottle remained sterile. All bacteria were incubated overnight with *E. coli* at 37°C and the other bacteria and the control at 25°C.

The next day, bacterial absorbances were measured with a spectrophotometer and used with the equations in Table S1 to estimate the bacterial density in each broth. The eighteen experimental plates were seeded in six groups of three, one group per treatment. 5 × 10^7^ CFU of each bacterial species were deposited onto the plates and water added to bring the final volume up to 500 µl to ensure even spreading. For the three control plates, the volume of sterile broth deposited was equal to the largest volume of bacteria added for that replicate. The liquid was then spread in an even lawn across the plate and let dry in a vent hood.

After two weeks of starvation, worms were washed off of their plates and treated with 1% sodium dodecyl sulfate (SDS) on a rocker table for 30 min. This treatment kills all worms except those in the dauer stage (Cassada & Russell, 1975). The worms were washed with water four times to remove the SDS and the final volume reduced to about 2 ml. Three aliquots of a 1:100 dilution of these worms were scanned for live worms to estimate live dauer density in the undiluted tubes. 2,000 dauers were then deposited in the center of experimental plates which were air‐dried in a vent hood and then stored at room temperature. The total time of exposure from worm deposition to worm removal was three hours. We chose this time point because in previous work with N2, three hours was long enough for roughly half the population to recover and so would hopefully capture as much variation as possible (Cassada & Russell, 1975).

### 3.3 Worm counting

The volume of worms placed in the center of experimental plates also contained the bodies of worms killed during the SDS wash, but most of the live worms explored the rest of the plate during the three‐hour exposure. This central spot was cut out of the agar to leave only worms that were live at the time of deposition. Worms were then washed off each experimental plate, treated with 1% SDS for 30 min, and then washed four times with water to remove excess SDS. Ten 20 µl aliquots per experimental plate were spotted onto an empty plate. Worms were then visually assayed for movement and given a maximum of three seconds to move before being declared dead. Moving worms were counted as having survived the SDS treatment, indicating that they had remained in dauer during the three‐hour exposure. Worms that did not move were counted as having been killed by the SDS wash, indicating that they had begun to recover from the dauer stage.

### 3.4 Fecundity assay

Synchronized L1 larvae of all three worm strains were acquired by following standard bleaching protocols and hatching the eggs overnight (Stiernagle, 2006). Populations of L1 larvae were spotted onto 60‐mm NGM plates with either no bacteria (the negative control) or 100 µl of overnight bacterial cultures. These plates were maintained at room temperature and scanned periodically for the presence of eggs and the general health of the population. The assay was done in triplicate.

### 3.5 Statistical analysis

Logistic regression models were built in R version 3.6.2. Several models were compared using the likelihood‐ratio test (Hosmer & Lemeshow, 2000). We retained all variables in the model because removing any of them significantly reduced the model's fit. Because worm strains had unique patterns of recovery, we also introduced an interaction term between the variables “Worm Strain” and “Treatment” and retained it in the model because it significantly increased the model's fit.

### 3.6 Bacterial genome sequencing

Overnight cultures of each bacterial isolate were grown at 25°C, with the exception of *E. coli* OP50 which was grown at 37 º C; one mL of each culture was place in a 1.5‐ml tube and centrifuged to pellet the bacteria. Excess media was removed from the tube prior to gDNA extraction. Genomic DNA was extracted from each sample using a modified phenol–chloroform extraction (Green & Sambrook, 2017). One microgram of DNA from each sample was then prepared for multiplexed sequencing by attaching unique barcodes to each sample from the Oxford Nanopore Technologies (ONT) Native Barcoding Kit (EXP‐NBD104). Following ligation of the barcode sequences, the DNA from each sample was pooled in equimolar amounts and prepared for sequencing using the ONT Ligation Sequencing Kit (SQK‐LSK109). The multiplexed sample was sequenced on a R9.4.1 flow cell using a GridION X5 platform. The sequence data were demultiplexed and trimmed of barcode sequences using Porechop. Each genome was then assembled using Canu v1.8 (Koren et al., 2017). Assembled genomes were uploaded to the Microbial Genomes Atlas (MiGA) server and processed using the NCBI Prok pipeline to identify the closest relatives of the bacterial isolates used in this study (Rodriguez et al., 2018). ANI, AAI, and analysis of 16S rRNA gene sequences were used from the pipeline to assign genus classifications.

### 3.7 Nematode DNA extraction, sequencing and analysis

*C. elegans* JU1395 worms were grown on several 100 mm NGM plates seeded with *E. coli* to achieve large population sizes. Worms were washed from the plates using M9 buffer, bleached using standard procedures, and the eggs hatched overnight (Stiernagle, 2006). We pelleted the worms, removed the supernatant, and then flash‐froze the pellet with liquid nitrogen. We then extracted the genomic DNA using a modified phenol–chloroform isolation (modified from Green & Sambrook, 2017). gDNA fragments were size selected using the Short Read Eliminator Kit from Circulomics Inc. One microgram of DNA was used to create a sequencing library with the ONT Ligation Sequencing Kit (SQK‐SK109) and sequenced on a R9.4.1 RevD flow cell using a GridION X5 platform. Adapter sequences were removed using Porechop and the genome assembled using Canu v 1.9 (Koren et al., 2017). The genome was polished using Illumina paired‐end reads generated by the CeNDR project (Cook, Zdraljevic, Roberts, & Andersen, 2017) and the Pilon software package (Walker et al., 2014). We used the BUSCO software v4.0.5 to estimate genic completeness with the nematoda_odb10 dataset (Seppey et al., 2019). We used the gmap‐gsnap software (Wu & Nacu, 2010) to align the N2 dauer gene transcripts to the CB4856 and JU1395 genome sequences. Polymorphisms were identified with Samtools (Li et al., 2009) and Bcftools (Li, 2011).

## 4 RESULTS

### 4.1 Fecundity assay

When categorizing the bacterial species as “beneficial” or “detrimental,” Samuel et al. (2016) only performed fecundity assays using the N2 strain, so these categories may only reflect one strain's growth ability. We expanded this assay to include CB4856 and JU1395 and found that they grow no differently than N2 on the range of bacteria, so the categorizations established in Samuel et al. (2016) hold. Worms on beneficial bacteria reached adulthood and produced eggs somewhere between 50 and 70.5 hr after they began feeding (Figure S2). *Serratia* sp. JUb9 attracted and killed worms such that the population could not progress past the first few larval stages. *Pseudomonas* sp. BIGb0427 repelled worms, keeping them in the first larval stage (L1) or the dauer stage. A few individuals managed to reach adulthood on the *Pseudomonas* sp. BIGb0427 plates, but only after feeding on small contaminants outside the lawn; the same phenomenon also occurred on control plates in the fecundity assay. Contaminants were not an issue in the dauer recovery assay where the lawn was spread evenly across the plate.

### 4.2 Dauer recovery assay

**Figure 2:**
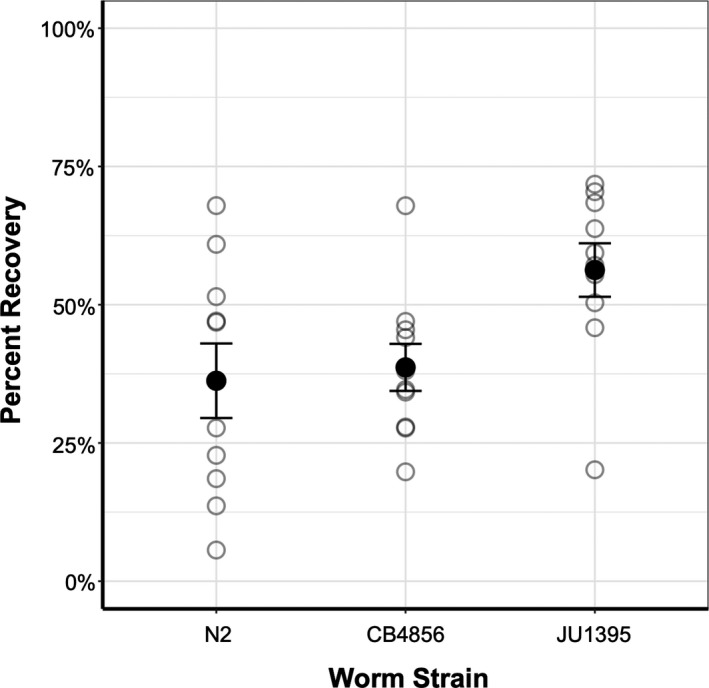
Mean recovery for the three worm strains. Faded points are average recovery values for each trial with all treatments combined. Error bars show standard error of the mean

Observations are summarized in Table 1. Of the 19,071 worms observed in this project, 8384 (or about 44%) recovered from the dauer stage after a three‐hour exposure. Recovery was not evenly distributed among the worm strains. N2 worms recovered the least—about 34.4%—which is consistent with previous work on recovery in this strain (Cassada & Russell, 1975). CB4856 had a slightly higher recovery at 39.2%, while JU1395 had a much higher recovery at 56.4% (Figure 2). Additionally, there were some batch effects among the trials; the worms in certain trials had depressed or enhanced recovery across the board (Figure S1).

**Table 1:** Summary of observations categorized by worm strain, bacterial treatment, and recovery status

	Control	*E. coli*	*Raoultella*	*Providencia*	*Pseudomonas*	*Serratia*
N2						
Total Worms	654	808	980	921	987	1,372
% Recovered	29.2	38.0	36.0	36.3	33.4	32.9
CB4856						
Total Worms	1,011	954	1,258	895	896	1,438
% Recovered	32.6	42.6	40.2	36.2	37.6	43.4
JU1395						
Total Worms	1,048	1,031	1,374	1,125	1,112	1,207
% Recovered	50.6	52.5	56.8	66.7	53.3	57.7

Note

Each cell of “Total Worms” is data aggregated from 10 trials, each of which contains 10 technical replicates.

**Figure 3:**
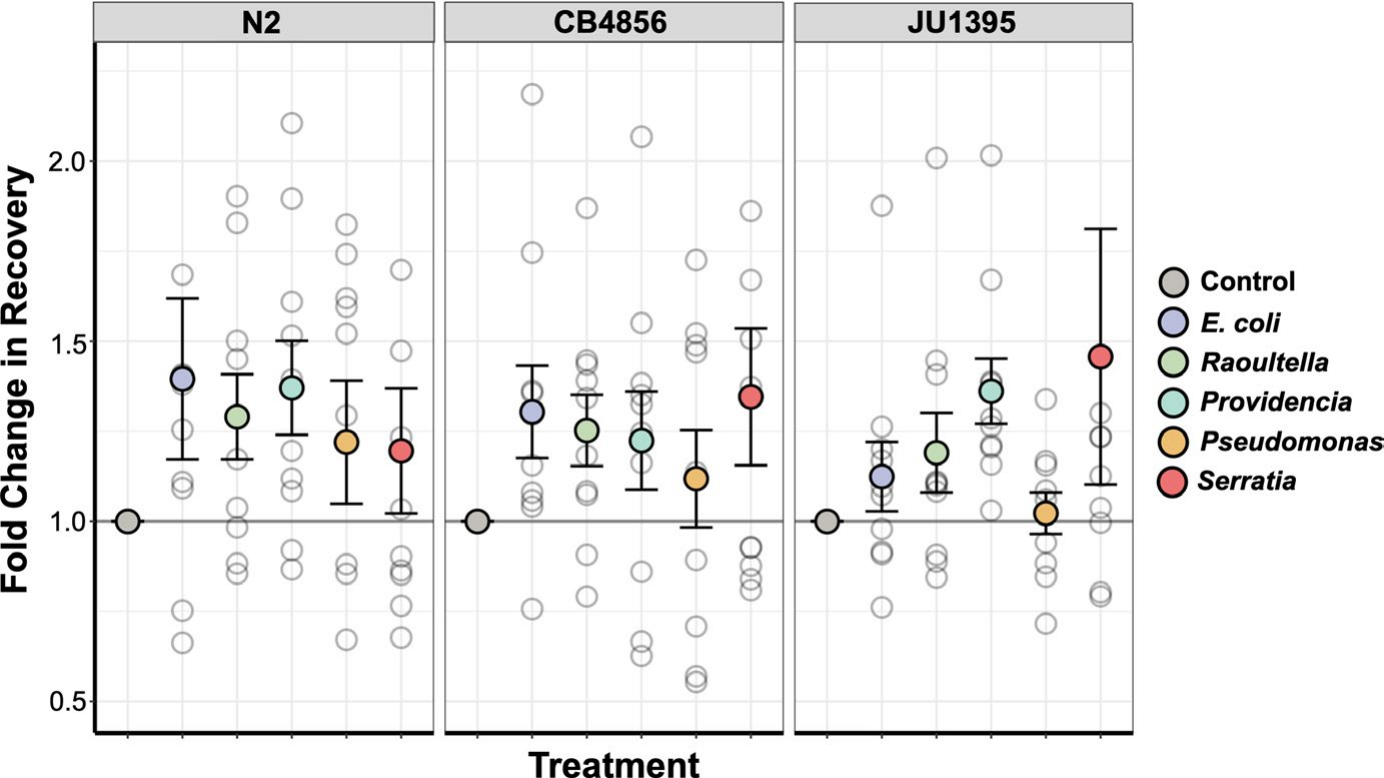
Fold change in recovery standardized by the percent recovered on the control of each trial. Cool colors represent beneficial bacteria and warm colors represent detrimental bacteria. Error bars show standard error of the mean. Five outlier points lie off the graph; Figure S3 is an expanded version of this figure which shows all points

Worm recovery depended on bacterial treatment but also on which strain was detecting the bacteria, suggesting an interaction between these two variables (Figure 3). N2 had broadly enhanced recovery on all beneficial bacteria with the highest mean recovery on *E. coli*. N2 also enhanced its recovery on the detrimental bacteria but only marginally. CB4856's recovery was similar to N2's but included an enhanced recovery on the detrimental bacterium *Serratia* sp. JUb9. JU1395 recovered the most on the beneficial bacterium *Providencia* sp. JUb39. JU1395's recovery on *Serratia* sp. JUb9 was also very high, although this seems driven by one outlier during trial 2 in which JU1395's recovery increased by a factor of 4.60.

### 4.3 Statistical analysis

Because recovering from dauer is a binary developmental choice, we built a logistic regression model to explore which variables affected a worm's probability of recovering. The basic results of the model are shown in Table 2. The model uses the worm strain N2 and the control treatment as baselines. Odds ratios represent the fold change in probability of recovering compared to the baseline. For example, any worm recovering on *E. coli* as opposed to the control has a 1.70‐fold increased probability of recovering. Odds ratios for the remaining variables can be found in Table S2.

**Table 2:** Estimated odds ratios for each value of the variables “Worm Strain” and “Treatment”

Variable	Value	Odds Ratio	95% CI
Worm Strain	N2	1.00	
	CB4856	1.28	(1.02, 1.59)
	JU1395	2.60	(2.10, 3.22)
Treatment	Control	1.00	
	*E. coli*	1.70	(1.34, 2.15)
	*Raoultella*	1.79	(1.38, 2.32)
	*Providencia*	1.50	(1.20, 1.88)
	*Pseudomonas*	1.54	(1.20, 1.97)
	*Serratia*	1.50	(1.17, 1.93)

Our model shows a significant interaction between “Worm Strain” and “Treatment”. This means that the odds ratios listed under “Treatment” in Table 2 should vary with worm strain. Table 3 shows the amounts by which they are adjusted, as well as the resulting odds ratios. Because N2 is the baseline worm strain and the control is the baseline treatment, N2 needs no adjustments nor do any of the controls. The adjustments are made to the original odds ratios by simple multiplication. For example, a worm's probability of recovery is predicted to increase 1.70‐fold when exposed to *E. coli*. CB4856, however, is 0.92 times less likely to recover on *E. coli* than N2, the baseline worm strain. Therefore, CB4856's recovery on *E. coli* is actually only 1.56‐fold higher than its recovery on the control.

**Table 3:** Odds ratios of treatments adjusted due to interactions between “Worm Strain” and “Treatment”

Worm strain	Treatment	Odds ratio (without interaction)	Odds ratio adjustment	Odds ratio (with interaction)
N2	Control	1.00		1.00
	*E. coli*	1.70		1.70
	*Raoultella*	1.79		1.79
	*Providencia*	1.50		1.50
	*Pseudomonas*	1.54		1.54
	*Serratia*	1.50		1.50
CB4856	Control	1.00		1.00
	*E. coli*	1.70	0.92	1.56
	*Raoultella*	1.79	0.86	1.53
	*Providencia*	1.50	0.79	1.18
	*Pseudomonas*	1.54	0.90	1.38
	*Serratia*	1.50	1.06	1.58
JU1395	Control	1.00		1.00
	*E. coli*	1.70	0.75	1.28
	*Raoultella*	1.79	0.85	1.52
	*Providencia*	1.50	1.32	1.98
	*Pseudomonas*	1.54	0.74	1.14
	*Serratia*	1.50	1.09	1.64

### 4.4 Bacteria sequencing

The results of our sequencing are shown in Table 4. We found that all of the wild bacteria except *Providencia* sp. JUb39 were closely related to previously reported genomes, albeit in unnamed species. We also found that the isolate JUb54, which was called *Enterobacter* sp. JUb54 in Samuel et al. (2016), may actually belong to the genus *Raoultella* based on analysis of the 16S rRNA gene sequence as well as Average Nucleotide Identity (ANI) and Average Amino Acid Identity (AAI); the use of *Raoultella* instead of *Enterobacter* is reflected in this article. Interestingly, *Serratia* sp. JUb9—which was found associated with *C. elegans* in France (Samuel et al., 2016)—is closely related to an isolate that was found in *C. elegans* habitats in Germany (Accession number: CP023268).

**Table 4:** Summary of information about sequenced bacteria

Species	Category	Genome size	Number of contigs
*Escherichia coli* OP50	Beneficial	4,616,404	1
*Raoultella* sp. JUb54	Beneficial	5,422,632	1
*Providencia* sp. JUb39	Beneficial	4,340,164	2
*Pseudomonas* sp. BIGb0427	Detrimental	5,864,124	7
*Serratia* sp. JUb9	Detrimental	5,108,081	1

### 4.5 Dauer genes

*C. elegans* dauer entry and recovery are influenced by several well‐characterized pathways including those underlying pheromone synthesis, guanylyl cyclase, TGFβ‐like, insulin‐like, and steroid hormone synthesis (Girard et al., 2007). Since the three worm strains responded differently to the range of bacteria, we sought to characterize molecular polymorphisms in these conserved dauer‐controlling pathways. N2 and CB4856 already had sequenced and assembled genomes (Kim et al., 2019), so we sequenced JU1395's genome to allow for comparisons between the three strains. The assembled sequence was 103,053,620 nucleotides in 161 contiguous pieces. We used the software BUSCO to estimate the completeness of the assembled sequence by searching for a set of 3,131 genes thought to be conserved across nematodes (Seppey, Manni, & Zdobnov, 2019). We identified 98% of these genes in our assembled sequence with 97.4% found in complete single copy, 0.6% duplicated, 0.5% fragmented, and 1.5% missing. For reference, the N2 *C. elegans* assembled genome sequence has 98.5% of this 3,131 gene set with 98% in single copy, 0.5% duplicated, 0.3% fragmented, and 1.2% missing.

We aligned 113 *C. elegans* transcripts from 67 dauer‐associated genes to the assembled CB4856 and JU1395 sequences. Neither genome has been fully annotated for protein‐coding genes, and we used these alignments to measure polymorphisms and potential divergence in genes underlying these pathways. We identified relatively few polymorphisms in these sequences in JU1395 and CB4856. For example, there were only 18 polymorphisms in 9 genes between N2 and JU1395 and 46 polymorphisms in 15 genes between N2 and CB4856. The full list of dauer‐associated pathways, genes, and polymorphisms is given in the appendix. These polymorphisms are interesting targets for future studies investigating the genetic basis of the worm–microbe interactions.

## 5 COMPETING INTERESTS

The authors declare that there are no conflicts of interest.

## AUTHOR CONTRIBUTION

**Louis Bubrig:** Conceptualization (lead); Data curation (lead); Formal analysis (lead); Investigation (lead); Methodology (lead); Writing‐original draft (lead); Writing‐review & editing (lead). **John Sutton:** Data curation (supporting); Formal analysis (supporting); Investigation (supporting); Methodology (supporting); Software (supporting); Writing‐original draft (supporting); Writing‐review & editing (supporting). **Janna Fierst:** Data curation (supporting); Formal analysis (supporting); Methodology (supporting); Project administration (lead); Software (supporting); Supervision (lead); Writing‐original draft (supporting); Writing‐review & editing (supporting).

## Supporting information

Fig S1

Fig S2

Fig S3

Table S1

Table S2

Supplementary Material

## Data Availability

DNA sequence data generated during this project have been deposited with the National Center for Biotechnology Information under Bioproject PRJNA622250 for JU1395 and PRJNA622270 for the microbial samples. Data is archived through the Dryad data repository (https://doi.org/10.5061/dryad.k6djh9w4j).
