# Supplementary figures and images for: Caenorhabditis elegans dauers vary recovery in response to bacteria from natural habitat
Source: Ecol Evol. 2020 Aug 24;10(18):9886–95. doi: 10.1002/ece3.6646 (PMC7520223; doi:10.1002/ece3.6646)

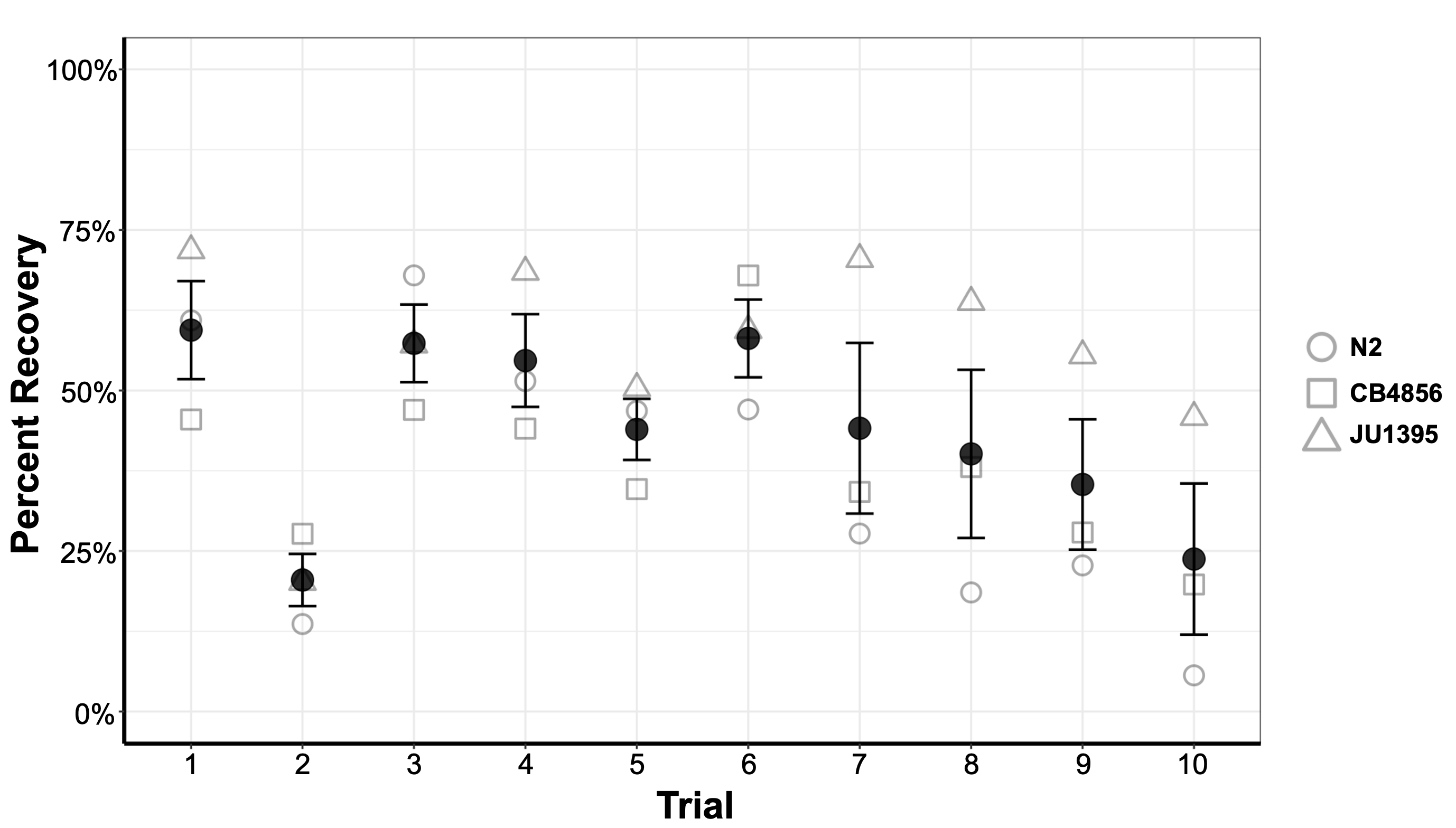

Supplement: Supplementary file 1 — Fig S1 [file ECE3-10-9886-s001.png]

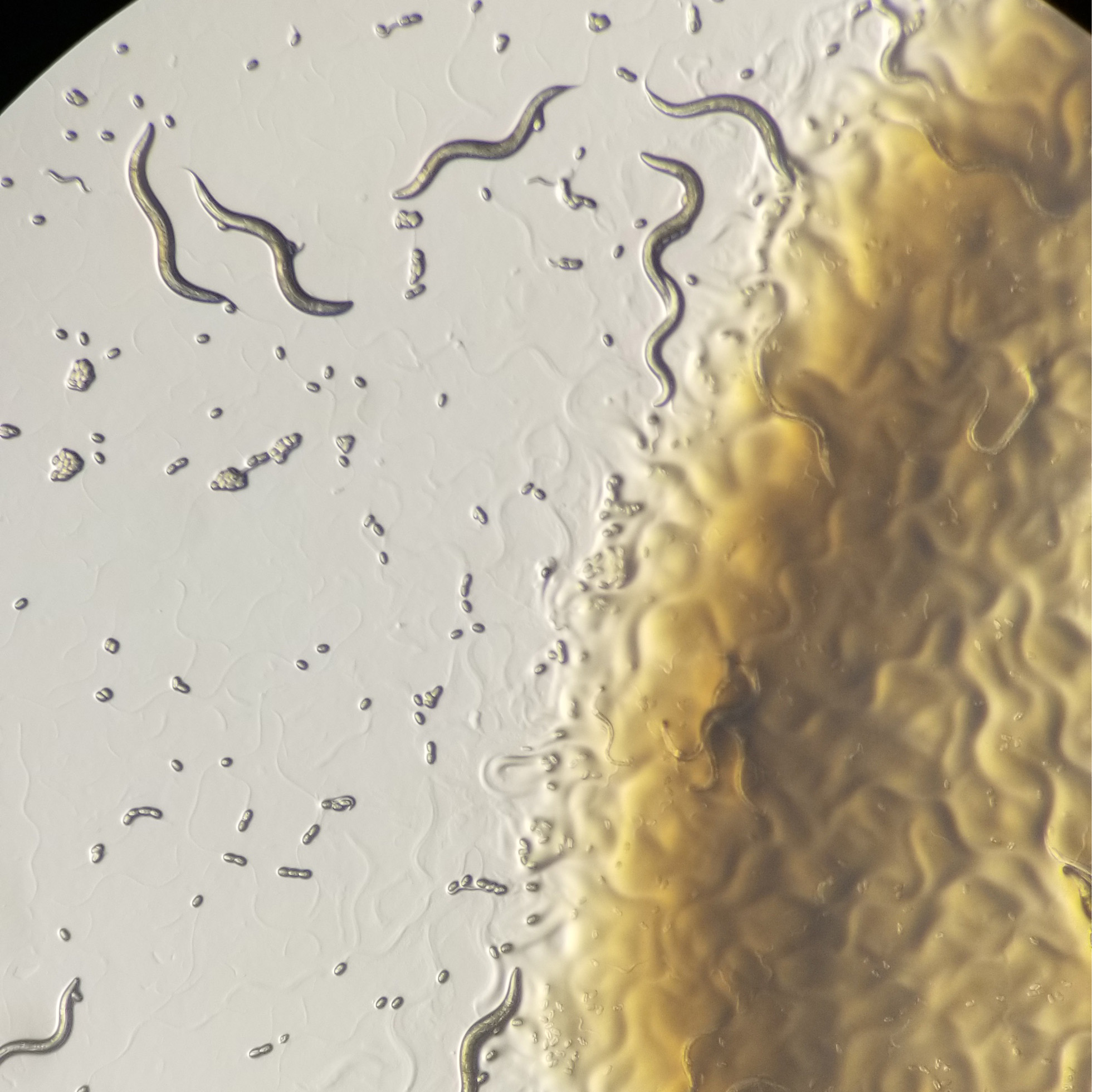

Supplement: Supplementary file 2 — Fig S2 [file ECE3-10-9886-s002.jpg]

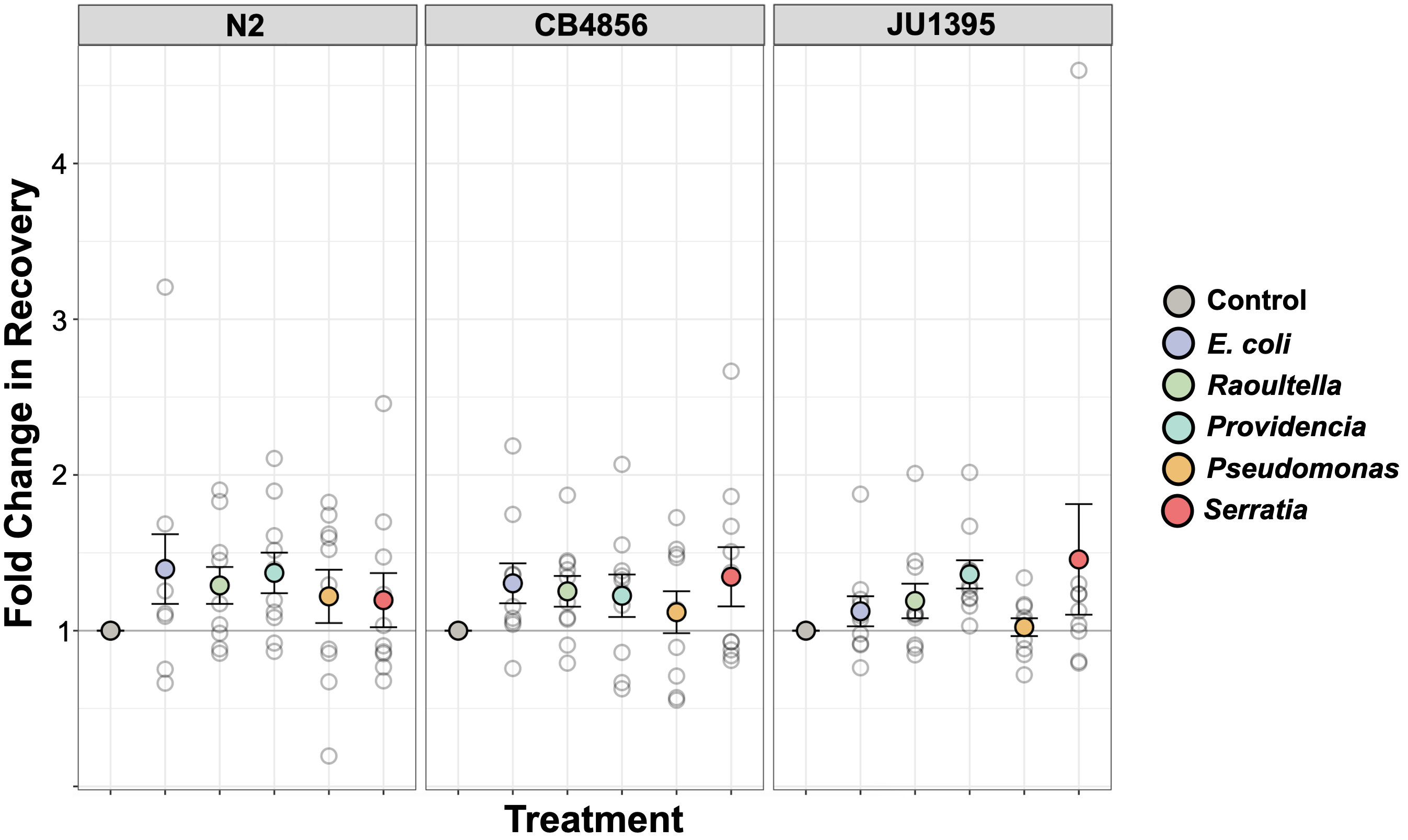

Supplement: Supplementary file 3 — Fig S3 [file ECE3-10-9886-s003.png]

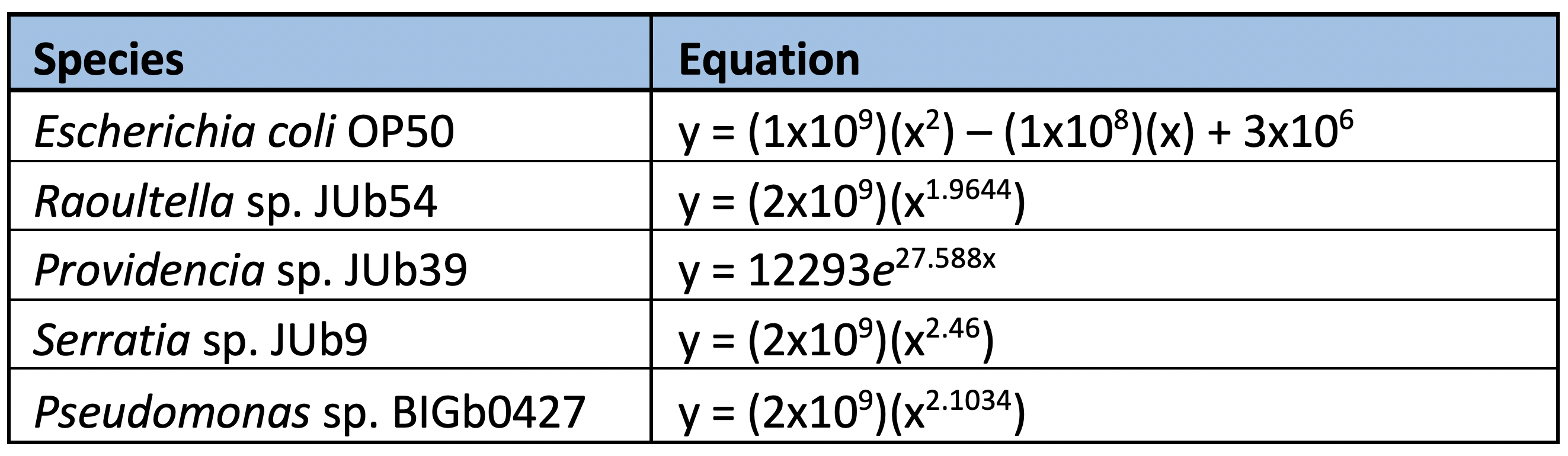

Supplement: Supplementary file 4 — Table S1 [file ECE3-10-9886-s004.png]

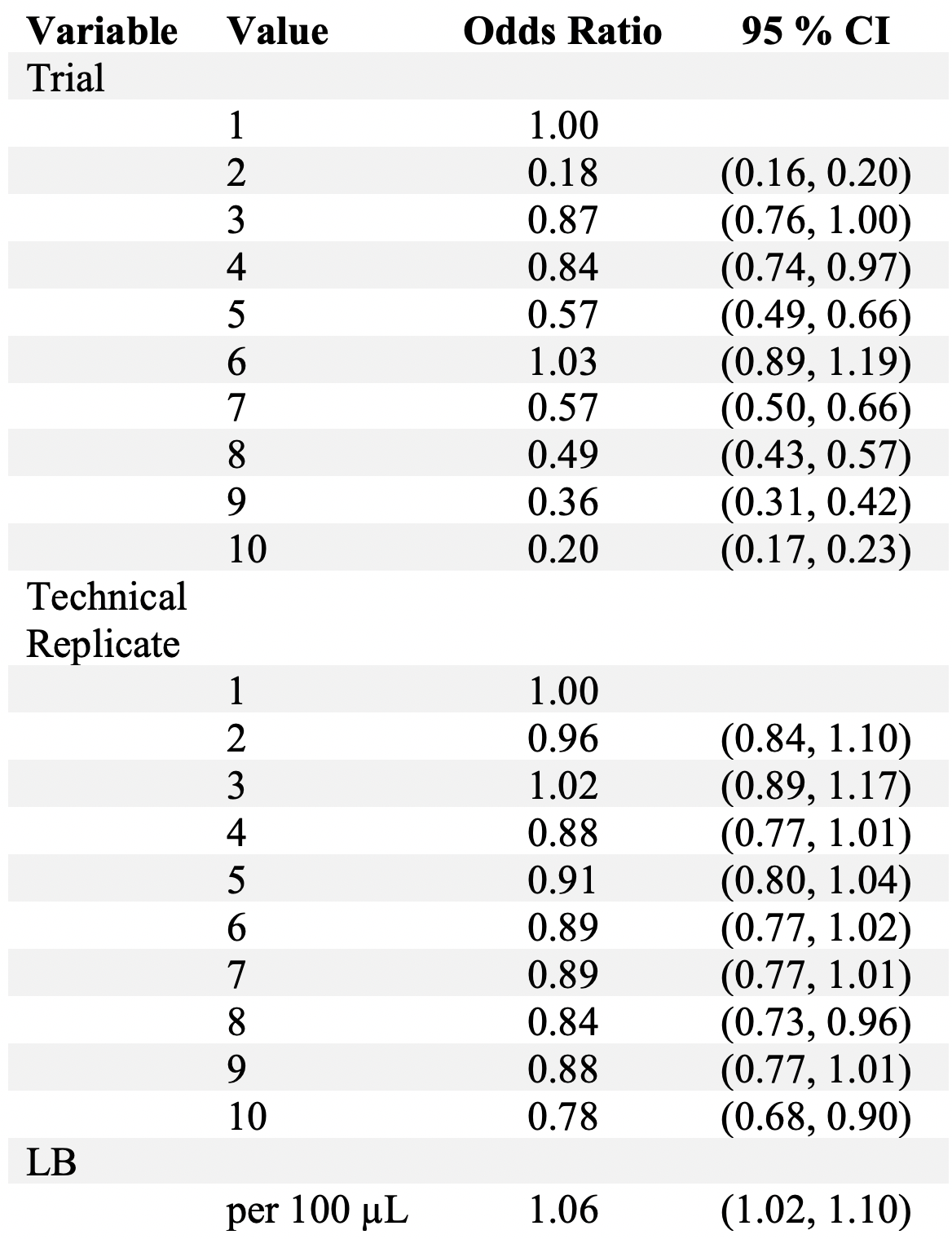

Supplement: Supplementary file 5 — Table S2 [file ECE3-10-9886-s005.png]
