# Supplementary material for: Caenorhabditis elegans dauers vary recovery in response to bacteria from natural habitat
Source: Ecol Evol. 2020 Aug 24;10(18):9886–95. doi: 10.1002/ece3.6646 (PMC7520223; doi:10.1002/ece3.6646)
Supplement: Supplementary file 6 — Supplementary Material [file ECE3-10-9886-s006.docx]

**Appendix**


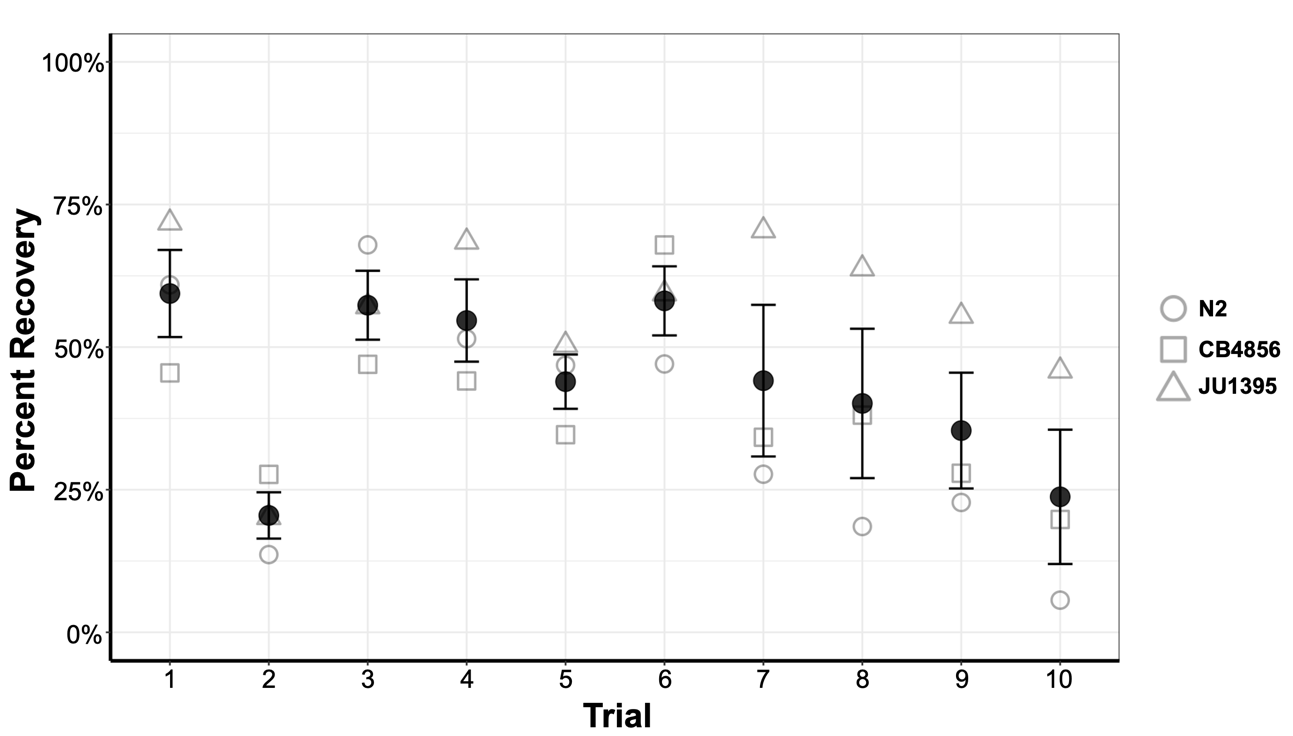


**Figure A1**. Mean recovery across the ten trials. Faded shapes are average values for each worm strain. Error bars show standard error of the mean.


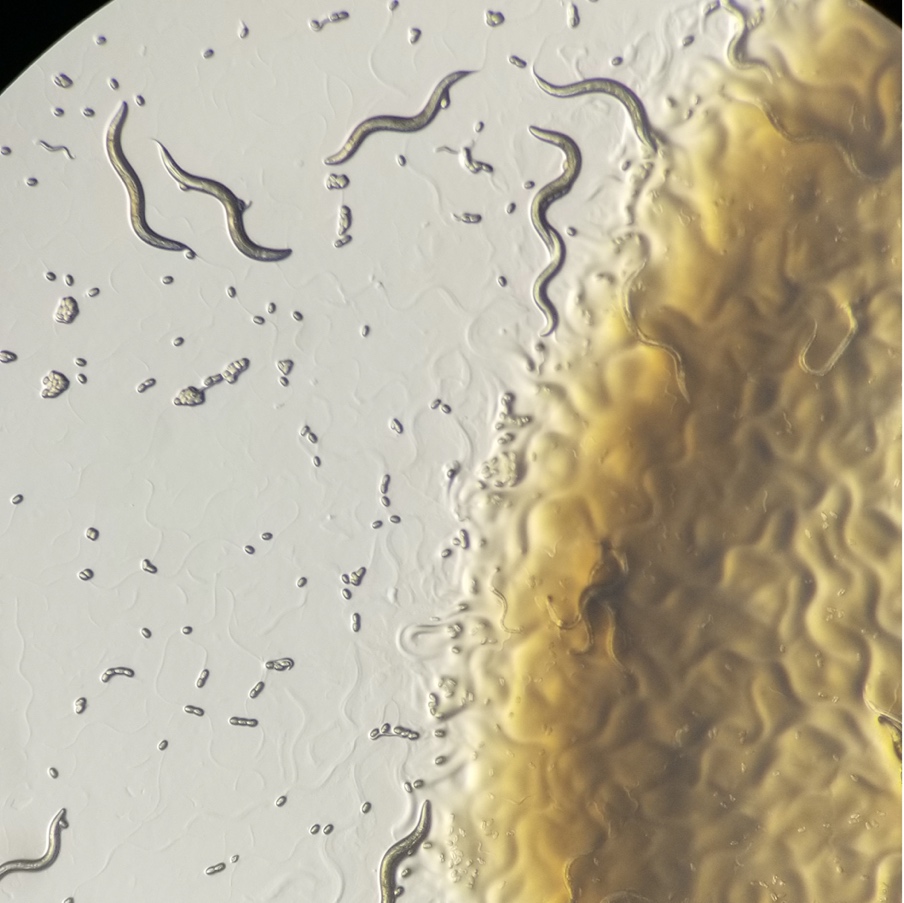


**Figure A2**. Worms of all three strains can establish populations on the beneficial bacteria


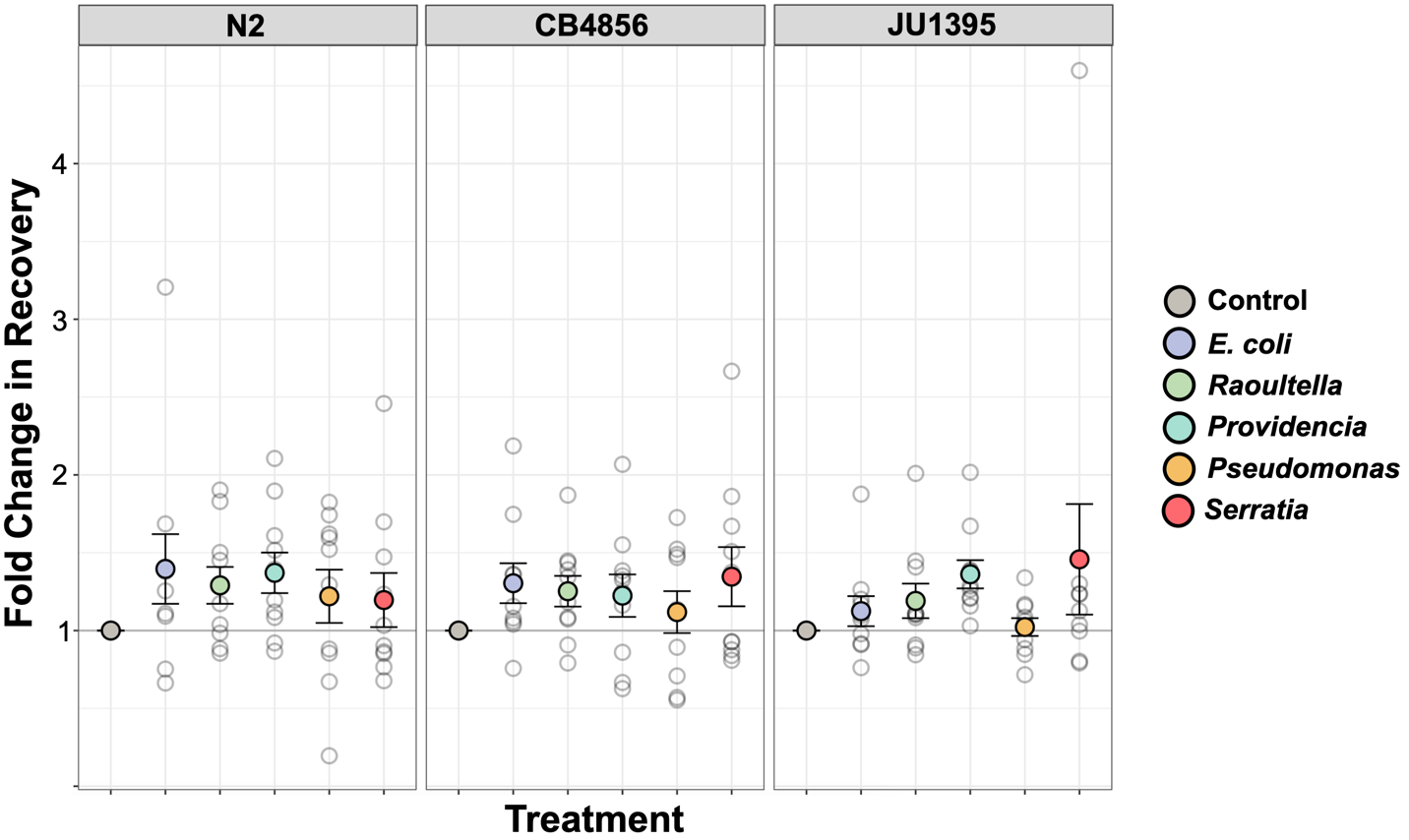


**Figure A3.** Expanded version of Figure 3 showing all points.

**Table A1**. Equations used to convert absorbance to bacterial density where *x* is the absorbance and *y* is CFU/mL.


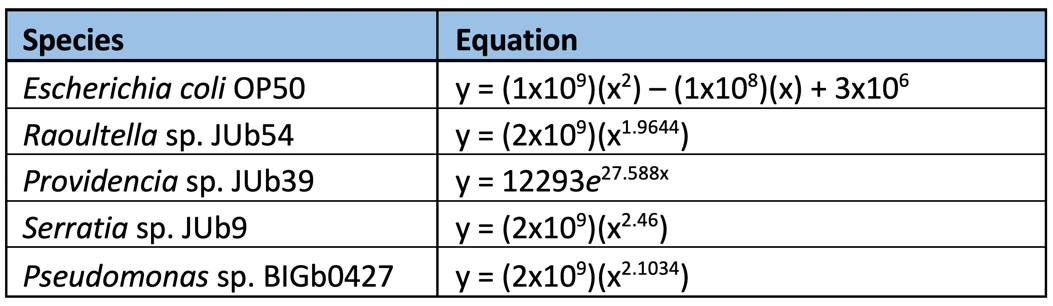


**Table A2**. Estimated odds ratios for each value of the variables "Trial," "Technical Replicate," and "LB".


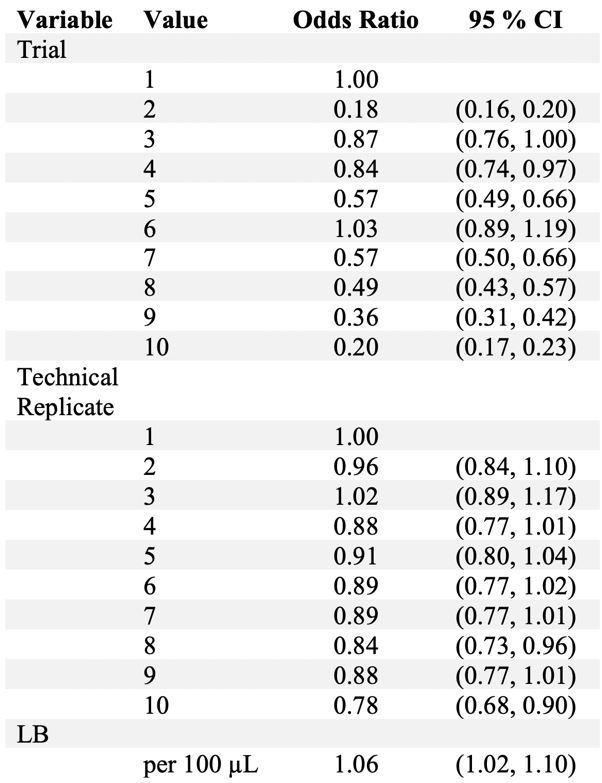


**Table A3**. *C. elegans* dauer genes

Pheromone synthesis:

*daf-22*

Guanylyl cyclase pathway:

*daf-11*

*tax-2*

*tax-4*

*daf-21*

TGFβ-like pathway:

*daf-1*

*daf-3*

*daf-4*

*daf-5*

*daf-7*

*daf-8*

*daf-14*

*scd-1*

*scd-2*

*scd-3*

*egl-4*

*bra-1*

*kin-8*

Insulin-like pathway:

*daf-2*

*daf-23*

*daf-16*

*ins-1*

*ins-2*

*…* through

*ins-40*

Steroid hormone pathway:

*daf-9*

*daf-12*

*ncr-1*

*ncr-2*

*Serratia* interactions:

*tol-1*

**Table A4**. *C. elegans* dauer gene transcripts

NM_001025812.3 Caenorhabditis elegans TOLl (Drosophila) family (tol-1), partial mRNA

NM_001025977.3 Caenorhabditis elegans Serine/threonine-protein kinase receptor (daf-4), partial mRNA

NM_001025978.2 Caenorhabditis elegans Receptor protein serine/threonine kinase (daf-4), partial mRNA

NM_001026422.4 Caenorhabditis elegans Forkhead box protein O (daf-16), partial mRNA

NM_001026423.4 Caenorhabditis elegans Forkhead box protein O (daf-16), partial mRNA

NM_001026424.4 Caenorhabditis elegans Forkhead box protein O (daf-16), partial mRNA

NM_001026425.3 Caenorhabditis elegans Forkhead box protein O (daf-16), partial mRNA

NM_001026426.2 Caenorhabditis elegans Forkhead box protein O (daf-16), partial mRNA

NM_001026427.4 Caenorhabditis elegans Forkhead box protein O (daf-16), partial mRNA

NM_001026675.1 Caenorhabditis elegans INSulin related (ins-29), partial mRNA

NM_001026676.1 Caenorhabditis elegans INSulin related (ins-27), partial mRNA

NM_001026678.1 Caenorhabditis elegans INSulin related (ins-25), partial mRNA

NM_001026679.1 Caenorhabditis elegans INSulin related (ins-28), partial mRNA

NM_001026791.2 Caenorhabditis elegans INSulin related (ins-13), partial mRNA

NM_001026792.3 Caenorhabditis elegans INSulin related (ins-12), partial mRNA

NM_001026793.1 Caenorhabditis elegans INSulin related (ins-38), partial mRNA

NM_001026982.1 Caenorhabditis elegans INSulin related (ins-14), partial mRNA

NM_001026983.1 Caenorhabditis elegans INSulin related (ins-15), partial mRNA

NM_001027168.1 Caenorhabditis elegans INSulin related (ins-19), partial mRNA

NM_001027358.4 Caenorhabditis elegans INSulin related (ins-20), partial mRNA

NM_001027670.1 Caenorhabditis elegans INSulin related (ins-16), partial mRNA

NM_001027988.4 Caenorhabditis elegans Cell surface receptor daf-1 (daf-1), partial mRNA

NM_001027989.3 Caenorhabditis elegans Cell surface receptor daf-1 (daf-1), partial mRNA

NM_001028052.2 Caenorhabditis elegans cGMP-dependent protein kinase egl-4 (egl-4), partial mRNA

NM_001028053.2 Caenorhabditis elegans cGMP-dependent protein kinase egl-4 (egl-4), partial mRNA

NM_001028954.1 Caenorhabditis elegans INSulin related (ins-10), partial mRNA

NM_001029191.1 Caenorhabditis elegans INSulin related (ins-9), partial mRNA

NM_001029376.4 Caenorhabditis elegans Nuclear hormone receptor family member daf-12 (daf-12), partial mRNA

NM_001029377.3 Caenorhabditis elegans Nuclear hormone receptor family member daf-12 (daf-12), partial mRNA

NM_001029378.1 Caenorhabditis elegans Nuclear hormone receptor family member daf-12 (daf-12), partial mRNA

NM_001029433.3 Caenorhabditis elegans Dwarfin sma (daf-3), partial mRNA

NM_001029434.2 Caenorhabditis elegans Dwarfin sma (daf-3), partial mRNA

NM_001029732.1 Caenorhabditis elegans Cytochrome P450 daf-9 (daf-9), partial mRNA

NM_001047774.2 Caenorhabditis elegans Nuclear hormone receptor family member daf-12 (daf-12), partial mRNA

NM_001264561.1 Caenorhabditis elegans Forkhead box protein O (daf-16), partial mRNA

NM_001264563.1 Caenorhabditis elegans Forkhead box protein O (daf-16), partial mRNA

NM_001264650.1 Caenorhabditis elegans INSulin related (ins-36), partial mRNA

NM_001264651.1 Caenorhabditis elegans INSulin related (ins-36), partial mRNA

NM_001268487.1 Caenorhabditis elegans INSulin related (ins-8), partial mRNA

NM_001268488.1 Caenorhabditis elegans INSulin related (ins-7), partial mRNA

NM_001268489.1 Caenorhabditis elegans Probable insulin-like peptide beta-type 4 (ins-7), partial mRNA

NM_001268546.1 Caenorhabditis elegans Uncharacterized protein (daf-14), partial mRNA

NM_001268547.1 Caenorhabditis elegans Uncharacterized protein (daf-14), partial mRNA

NM_001307520.1 Caenorhabditis elegans Uncharacterized protein (egl-4), partial mRNA

NM_001307521.1 Caenorhabditis elegans cGMP-dependent protein kinase (egl-4), partial mRNA

NM_001312987.1 Caenorhabditis elegans Receptor protein-tyrosine kinase (daf-2), partial mRNA

NM_001312988.1 Caenorhabditis elegans Receptor protein-tyrosine kinase (daf-2), partial mRNA

NM_001312989.1 Caenorhabditis elegans Receptor protein-tyrosine kinase (daf-2), partial mRNA

NM_001312990.1 Caenorhabditis elegans Uncharacterized protein (daf-2), partial mRNA

NM_001312991.1 Caenorhabditis elegans Uncharacterized protein (daf-2), partial mRNA

NM_001313082.1 Caenorhabditis elegans Uncharacterized protein (daf-11), partial mRNA

NM_001313412.1 Caenorhabditis elegans Dwarfin sma (daf-3), partial mRNA

NM_001313413.1 Caenorhabditis elegans Dwarfin sma (daf-3), partial mRNA

NM_001313414.1 Caenorhabditis elegans Dwarfin sma (daf-3), partial mRNA

NM_001313415.1 Caenorhabditis elegans Dwarfin sma (daf-3), partial mRNA

NM_001313416.1 Caenorhabditis elegans Dwarfin sma (daf-3), partial mRNA

NM_001313417.1 Caenorhabditis elegans Uncharacterized protein (daf-3), partial mRNA

NM_001313473.1 Caenorhabditis elegans Uncharacterized protein (daf-16), partial mRNA

NM_001313474.1 Caenorhabditis elegans Uncharacterized protein (daf-16), partial mRNA

NM_001313504.1 Caenorhabditis elegans Uncharacterized protein (daf-16), partial mRNA

NM_001313505.1 Caenorhabditis elegans Uncharacterized protein (daf-16), partial mRNA

NM_001322590.1 Caenorhabditis elegans Serine/threonine-protein kinase receptor (daf-4), partial mRNA

NM_001330884.1 Caenorhabditis elegans Receptor protein serine/threonine kinase (daf-4), partial mRNA

NM_059830.5 Caenorhabditis elegans INSulin related (ins-18), partial mRNA

NM_059920.3 Caenorhabditis elegans Dwarfin sma (daf-8), partial mRNA

NM_060026.5 Caenorhabditis elegans Uncharacterized protein (tax-2), partial mRNA

NM_060988.3 Caenorhabditis elegans INSulin related (ins-33), partial mRNA

NM_061042.5 Caenorhabditis elegans INSulin related (ins-24), partial mRNA

NM_061043.3 Caenorhabditis elegans INSulin related (ins-30), partial mRNA

NM_061044.4 Caenorhabditis elegans INSulin related (ins-26), partial mRNA

NM_062053.1 Caenorhabditis elegans INSulin related (ins-31), partial mRNA

NM_062254.1 Caenorhabditis elegans INSulin related (ins-32), partial mRNA

NM_062670.1 Caenorhabditis elegans B-chain-like peptide (ins-11), partial mRNA

NM_062793.1 Caenorhabditis elegans Probable insulin-like peptide beta-type 2 (ins-2), partial mRNA

NM_062794.5 Caenorhabditis elegans Probable insulin-like peptide beta-type 3 (ins-3), partial mRNA

NM_062795.1 Caenorhabditis elegans Probable insulin-like peptide beta-type 1 (ins-4), partial mRNA

NM_062796.4 Caenorhabditis elegans Putative insulin-like peptide beta-type 6 (ins-5), partial mRNA

NM_062797.1 Caenorhabditis elegans Probable insulin-like peptide beta-type 5 (ins-6), partial mRNA

NM_064238.3 Caenorhabditis elegans Non-specific lipid-transfer protein-like 2 (daf-22), partial mRNA

NM_064501.2 Caenorhabditis elegans INSulin related (ins-37), partial mRNA

NM_064540.5 Caenorhabditis elegans Uncharacterized protein (daf-5), partial mRNA

NM_064864.4 Caenorhabditis elegans Dauer larva development regulatory growth factor daf-7 (daf-7), partial mRNA

NM_065249.4 Caenorhabditis elegans Insulin-like receptor subunit beta (daf-2), partial mRNA

NM_065510.4 Caenorhabditis elegans INSulin related (ins-17), partial mRNA

NM_065810.5 Caenorhabditis elegans Cell surface receptor daf-4 (daf-4), partial mRNA

NM_066412.3 Caenorhabditis elegans Niemann-Pick C1 protein homolog 2 (ncr-2), partial mRNA

NM_066632.4 Caenorhabditis elegans Cyclic nucleotide-gated cation channel (tax-4), partial mRNA

NM_066641.4 Caenorhabditis elegans Suppressor of activated egl-4 protein 2 (saeg-2), partial mRNA

NM_066821.2 Caenorhabditis elegans Probable insulin-like peptide alpha-type 1 (ins-21), partial mRNA

NM_066822.3 Caenorhabditis elegans Probable insulin-like peptide alpha-type 2 (ins-22), partial mRNA

NM_066823.1 Caenorhabditis elegans Probable insulin-like peptide alpha-type 3 (ins-23), partial mRNA

NM_067740.4 Caenorhabditis elegans cGMP-dependent protein kinase egl-4 (egl-4), partial mRNA

NM_067741.3 Caenorhabditis elegans cGMP-dependent protein kinase egl-4 (egl-4), partial mRNA

NM_069525.4 Caenorhabditis elegans INSulin related (ins-1), partial mRNA

NM_070301.2 Caenorhabditis elegans INSulin related (ins-34), partial mRNA

NM_072284.3 Caenorhabditis elegans ALK tyrosine kinase receptor homolog scd-2 (scd-2), partial mRNA

NM_073368.7 Caenorhabditis elegans Suppressor of activated egl-4 protein 1 (saeg-1), partial mRNA

NM_073559.5 Caenorhabditis elegans Receptor-type guanylate cyclase daf-11 (daf-11), partial mRNA

NM_074225.3 Caenorhabditis elegans Heat shock protein 90 (daf-21), partial mRNA

NM_075525.3 Caenorhabditis elegans INSulin related (ins-35), partial mRNA

NM_075760.4 Caenorhabditis elegans Dwarfin sma (daf-3), partial mRNA

NM_075846.3 Caenorhabditis elegans INSulin related (ins-39), partial mRNA

NM_076370.3 Caenorhabditis elegans Niemann-Pick C1 protein homolog 1 (ncr-1), partial mRNA

NM_077876.3 Caenorhabditis elegans BMP Receptor Associated protein family (bra-1), partial mRNA

NM_171279.3 Caenorhabditis elegans cGMP-dependent protein kinase egl-4 (egl-4), partial mRNA

NM_171280.2 Caenorhabditis elegans cGMP-dependent protein kinase egl-4 (egl-4), partial mRNA

NM_171699.4 Caenorhabditis elegans Cytochrome P450 daf-9 (daf-9), partial mRNA

NM_171785.3 Caenorhabditis elegans Suppressor of Constitutive Dauer formation (scd-1), partial mRNA

NM_171974.4 Caenorhabditis elegans Suppressor of Constitutive Dauer formation (scd-1), partial mRNA

NR_131392.1 Caenorhabditis elegans Non-coding transcript of protein-coding gene ins-36 (ins-36), miscRNA

NR_131589.1 Caenorhabditis elegans Non-coding transcript of protein-coding gene ins-8 (ins-8), miscRNA

NR_132448.1 Caenorhabditis elegans Non-coding transcript of protein-coding gene daf-2 (daf-2), miscRNA

NR_132532.1 Caenorhabditis elegans Non-coding transcript of protein-coding gene daf-11 (daf-11), miscRNA

**Table A5**. *C. elegans* CB4856 dauer transcript polymorphisms

Contig Position ID Reference Alternate Transcript

CP038187.1 508877 . A G NM_001025812.3

CP038187.1 509442 . A G NM_001025812.3

CP038187.1 14409957 . C A NM_001026675.1,

NM_001026676.1,

NM_001026678.1,

NM_001026679.1

CP038187.1 14432590 . C T NM_001026675.1,

NM_001026676.1,

NM_001026678.1,

NM_001026679.1

CP038188.1 3211977 . C T NM_001027168.1

CP038188.1 3211984 . G A NM_001027168.1

CP038188.1 3212158 . C T NM_001027168.1

CP038188.1 3212167 . G A NM_001027168.1

CP038188.1 3946515 . C G NM_062254.1

CP038188.1 5920857 . A G NM_001026793.1

CP038188.1 5920858 . C T NM_001026793.1

CP038188.1 5934734 . G C NM_001026791.2

CP038188.1 6381928 . A C NM_062796.4

CP038188.1 12887591 . T C NM_064238.3

CP038188.1 14564758 . C T NM_064540.5

CP038188.1 14564773 . A G NM_064540.5

CP038188.1 14566793 . A G NM_064540.5

CP038189.1 868851 . G A NM_064864.4

CP038189.1 3241442 . T C NM_001312987.1,

NM_001312988.1,

NM_001312989.1,

NM_001312990.1,

NM_001312991.1,

NM_065249.4,

NR_132448.1

CP038189.1 3242621 . T A NM_001312987.1,

NM_001312988.1,

NM_001312989.1,

NM_001312990.1,

NM_001312991.1,

NM_065249.4,

NR_132448.1

CP038189.1 3243526 . C T NM_001312987.1,

NM_001312988.1,

NM_001312989.1,

NM_001312990.1,

NM_001312991.1,

NM_065249.4,

NR_132448.1

CP038189.1 3243758 . C G NM_001312987.1,

NM_001312988.1,

NM_001312989.1,

NM_001312990.1,

NM_001312991.1,

NM_065249.4,

NR_132448.1

CP038189.1 5916103 . C T NM_001025978.2,

NM_001322590.1,

NM_065810.5

CP038189.1 9451763 . G T NM_066632.4

CP038189.1 9511211 . T C NM_066641.4

CP038189.1 9511214 . T G NM_066641.4

CP038189.1 9511216 . T C NM_066641.4

CP038190.1 1858555 . T C NM_067741.3

CP038190.1 10369987 . G T NM_001268547.1

CP038190.1 10370717 . A G NM_001268547.1

CP038190.1 10371317 . G T NM_001268547.1

CP038191.1 6587736 . G A NM_072284.3

CP038191.1 6587962 . C T NM_072284.3

CP038191.1 6588499 . T C NM_072284.3

CP038191.1 6588730 . A G NM_072284.3

CP038191.1 6589213 . A T NM_072284.3

CP038191.1 6589572 . C T NM_072284.3

CP038191.1 6589592 . G T NM_072284.3

CP038191.1 6590394 . A G NM_072284.3

CP038191.1 11754638 . T A NM_001313082.1,

NM_073559.5,

NR_132532.1

CP038191.1 11755672 . T C NM_001313082.1,

NM_073559.5,

NR_132532.1

CP038192.1 849854 . T A NM_001029433.3,

NM_001029434.2,

NM_001313412.1,

NM_001313413.1,

NM_001313414.1,

NM_001313415.1,

NM_001313416.1,

NM_001313417.1,

NM_075760.4

CP038192.1 4528158 . G A NM_076370.3

CP038192.1 4531883 . T G NM_076370.3

CP038192.1 4532576 . A G NM_076370.3

CP038192.1 4533748 . G A NM_076370.3

**Table A6**. *C. elegans* JU1395 dauer transcript polymorphisms

Contig Position ID Reference Alternate Transcript

tig00000092 2423999 . A G NM_171785.3,

NM_171974.4

tig00000120 2019762 . A G NM_001029191.1

tig00000125 502781 . C T NM_001028052.2,

NM_001028053.2,

NM_001307520.1,

NM_001307521.1,

NM_067740.4,

NM_067741.3,

NM_171279.3,

NM_171280.2

tig00000125 514996 . T C NM_001028052.2,

NM_001028053.2,

NM_001307520.1,

NM_001307521.1,

NM_067740.4,

NM_067741.3,

NM_171279.3,

NM_171280.2

tig00000258 517417 . C T NM_001027168.1

tig00000258 517598 . C T NM_001027168.1

tig00000258 517607 . G A NM_001027168.1

tig00000258 517629 . T C NM_001027168.1

tig00000258 517630 . T C NM_001027168.1

tig00000383 222668 . G C NM_062254.1

tig00007769 2101054 . G A NM_064238.3

tig00007769 2101075 . G A NM_064238.3

tig00007769 2101237 . A G NM_064238.3

tig00007770 471905 . A G NM_001026793.1

tig00007770 471906 . C T NM_001026793.1

tig00007770 496385 . T C NM_001026792.3

tig00007778 854013 . A G NM_001029433.3,

NM_001029434.2,

NM_001313412.1,

NM_001313413.1,

NM_001313414.1,

NM_001313415.1,

NM_001313416.1,

NM_075760.4

tig00007778 855295 . A T NM_001029433.3,

NM_001029434.2,

NM_001313412.1,

NM_001313413.1,

NM_001313414.1,

NM_001313415.1,

NM_001313416.1,

NM_075760.4
